# Supplementary material for: Principles for enhancing virus capsid capacity and stability from a thermophilic virus capsid structure
Source: Nat Commun. 2019 Oct 2;10:4471. doi: 10.1038/s41467-019-12341-z (PMC6775164; doi:10.1038/s41467-019-12341-z)
Supplement: Supplementary file 1 — Supplementary Information [file 41467_2019_12341_MOESM1_ESM.pdf]

SUPPLEMENTARY INFORMATION

"Principles for enhancing virus capsid capacity and stability from a thermophilic virus capsid structure"

Stone et al.

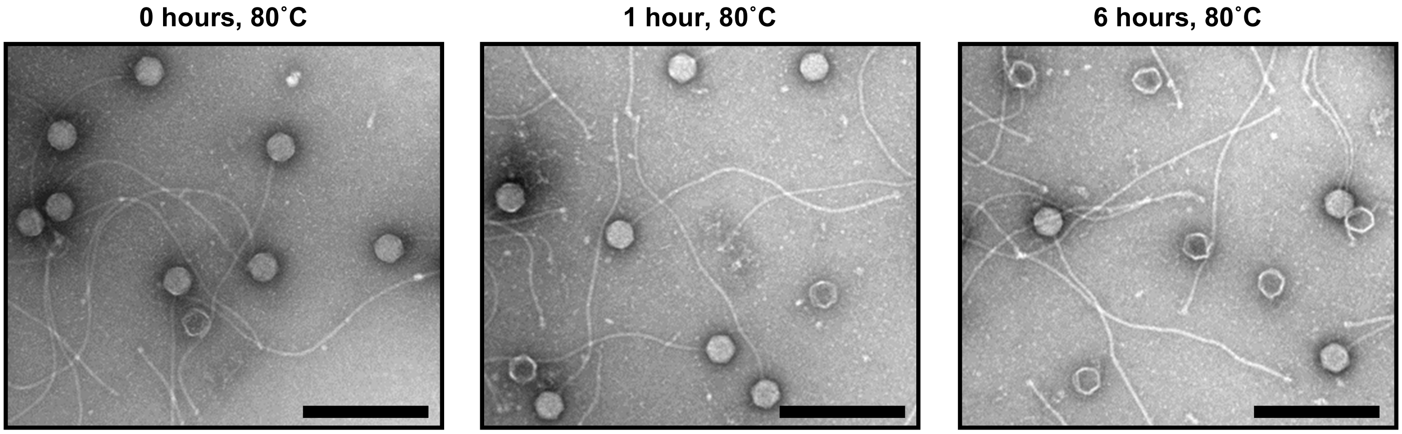

Supplementary Figure 1. Thermostability of P74-26 virions.
Negative-staining electron micrographs of purified P74-26 virions following incubation at 80˚C, imaged after 0, 1, and 6 hour incubations (see main text figure 1); scale bars, 500 nm.

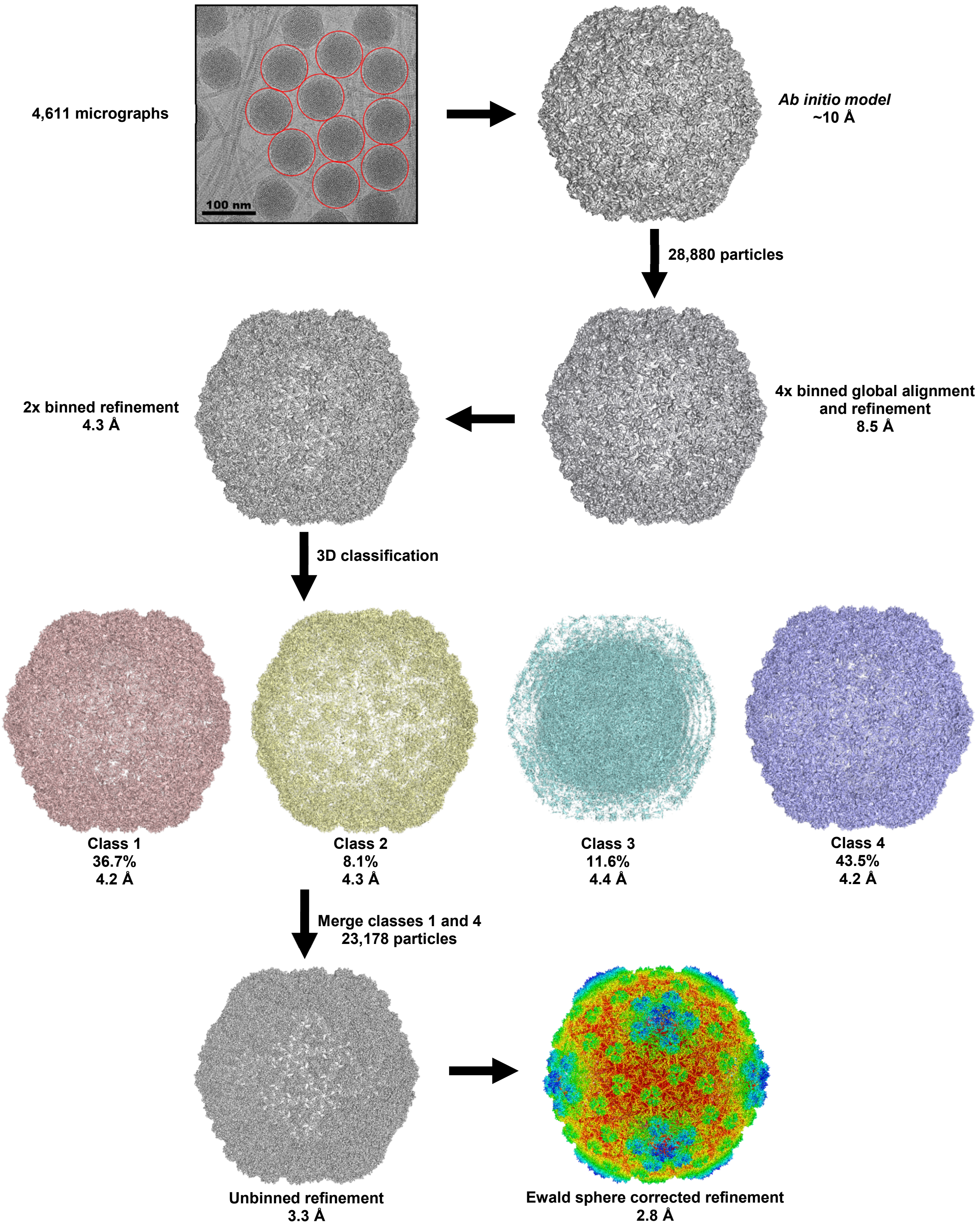

Supplementary Figure 2. Schematic of P74-26 capsid cryo-EM refinement and classification.
All particles were initially aligned to a single *ab initio* model generated from the data. 3D classification was performed with the 2x binned particle stack, and classes 1 and 4 were merged prior to final refinement of the capsid. Ewald sphere curvature correction was performed on the final round of refinement.

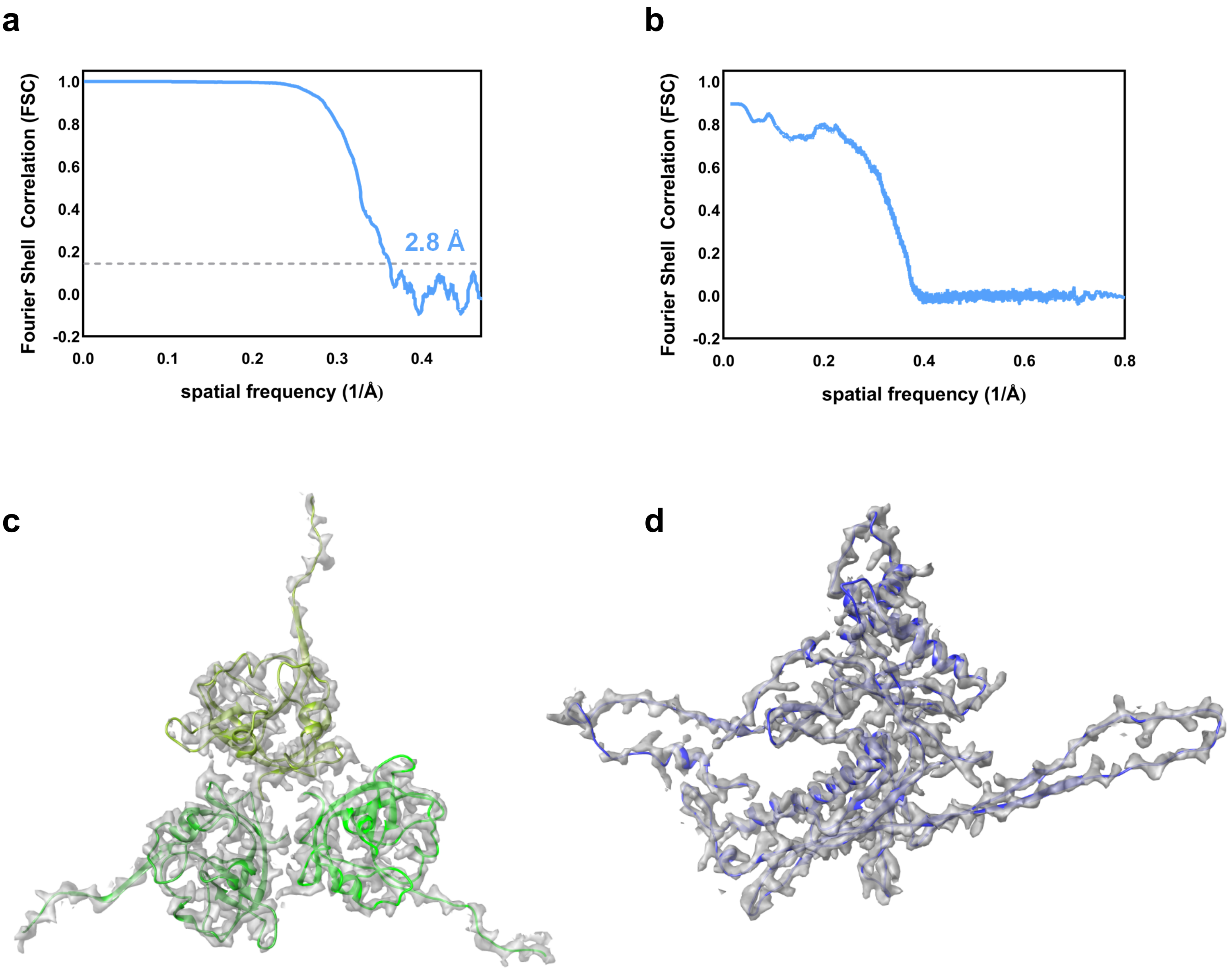

Supplementary Figure 3. P74-26 icosahedral reconstruction structure validation.
a) Fourier shell correlation curve for the P74-26 icosahedral reconstruction; resolution is 2.8 Å at the gold standard FSC cutoff of 0.143 (dotted gray line).
b) Fourier shell correlation curve for the comparison of the cryo-EM map to the coordinate model of the icosahedral asymmetric unit.
c) Model of the $Dec^{p74\text{-}26}$ protein trimer fit into the corresponding map density.
d) Overall fit of the P74-26 MCP monomer model fitted to the corresponding map density.

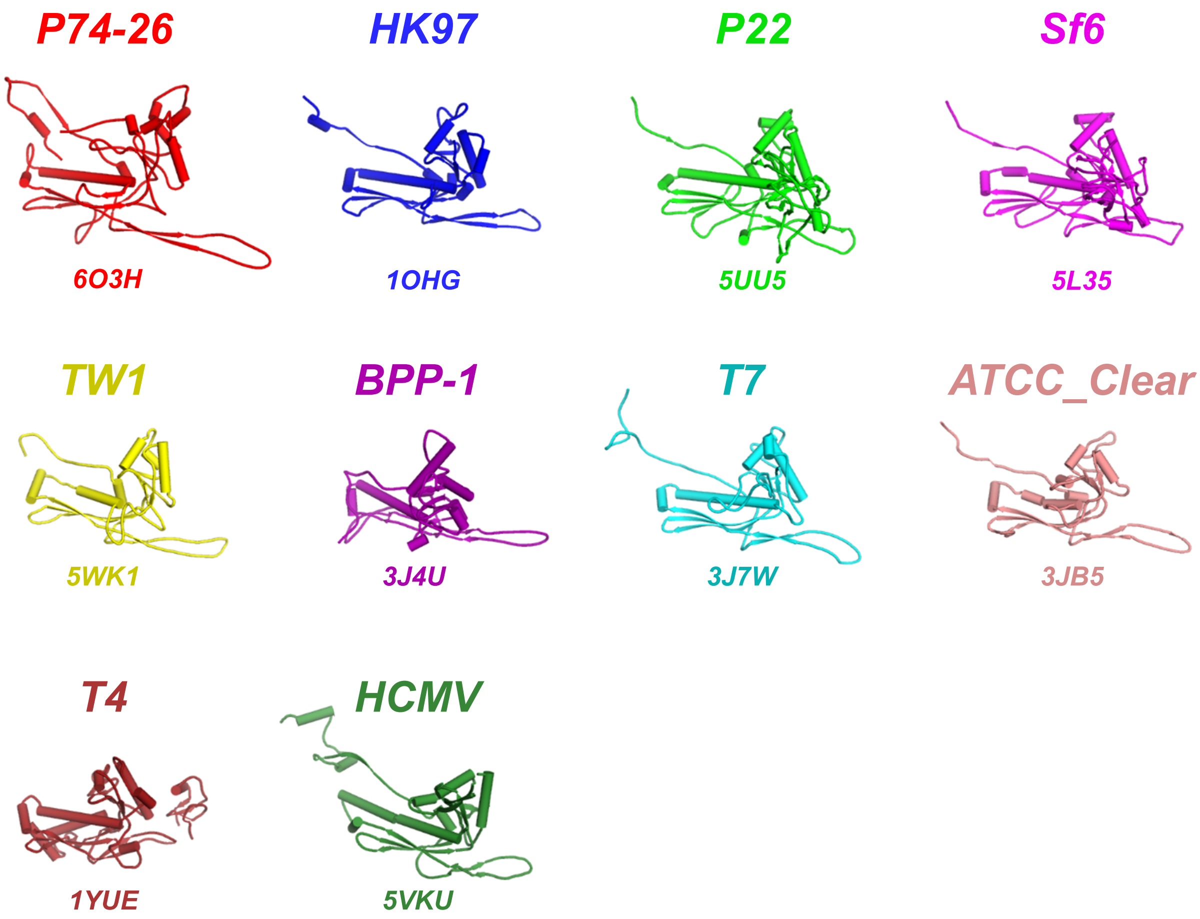

Supplementary Figure 4. Comparison of Major Capsid Protein Johnson folds. Johnson folds of MCP from P74-26, related Caudoviruses, and the conserved Johnson fold domain of Human Cytomegalovirus (HCMV) MCP. PDB accession codes are listed below each model.

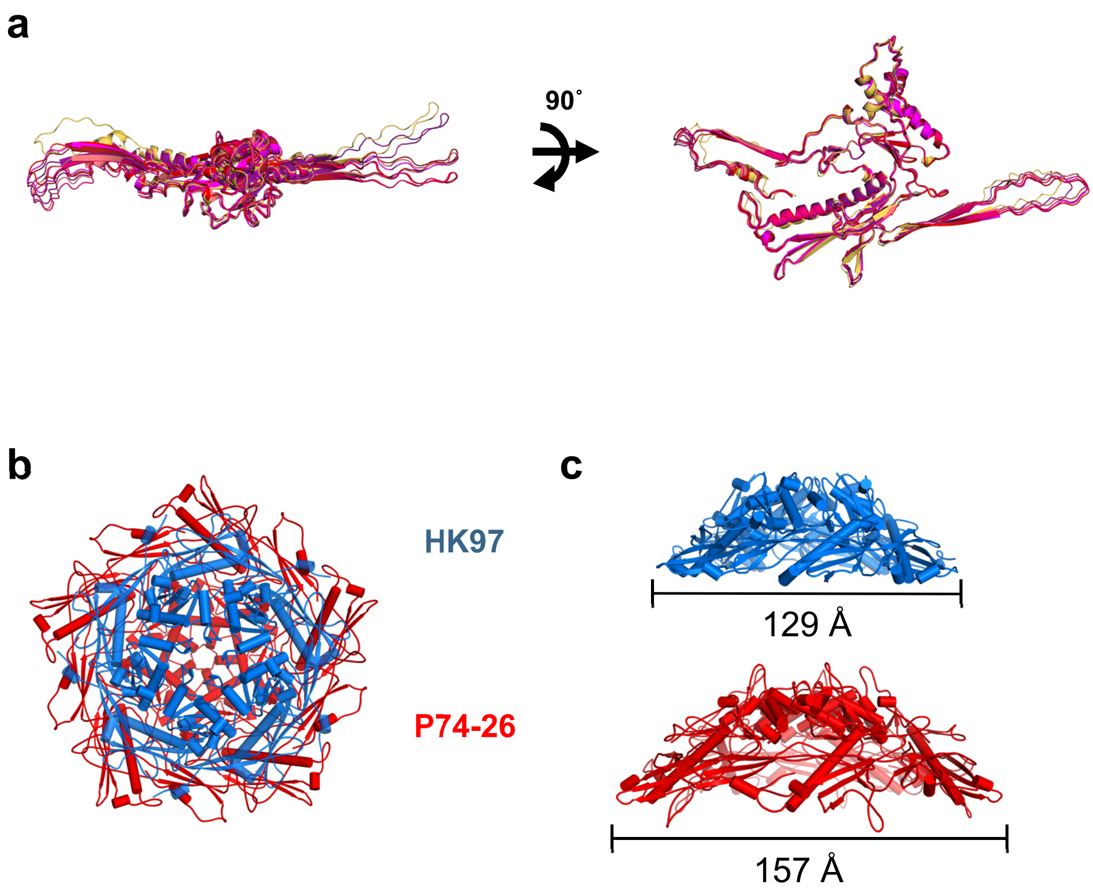

Supplementary Figure 5. MCP subunit and capsomer orientation in P74-26.
a) Overlay of MCP subunits in the P74-26 asymmetric unit shows the conformational heterogeneity of the E-loop and N-arm lassos, and the conserved orientation of the A- and P-domains.
b) Overlay of P74-26 (red) and phage HK97 (blue) pentons displayed as a top-down view.
c) Side profile comparison of P74-26 and HK97 pentons, values indicate longest diameter of respective pentons.

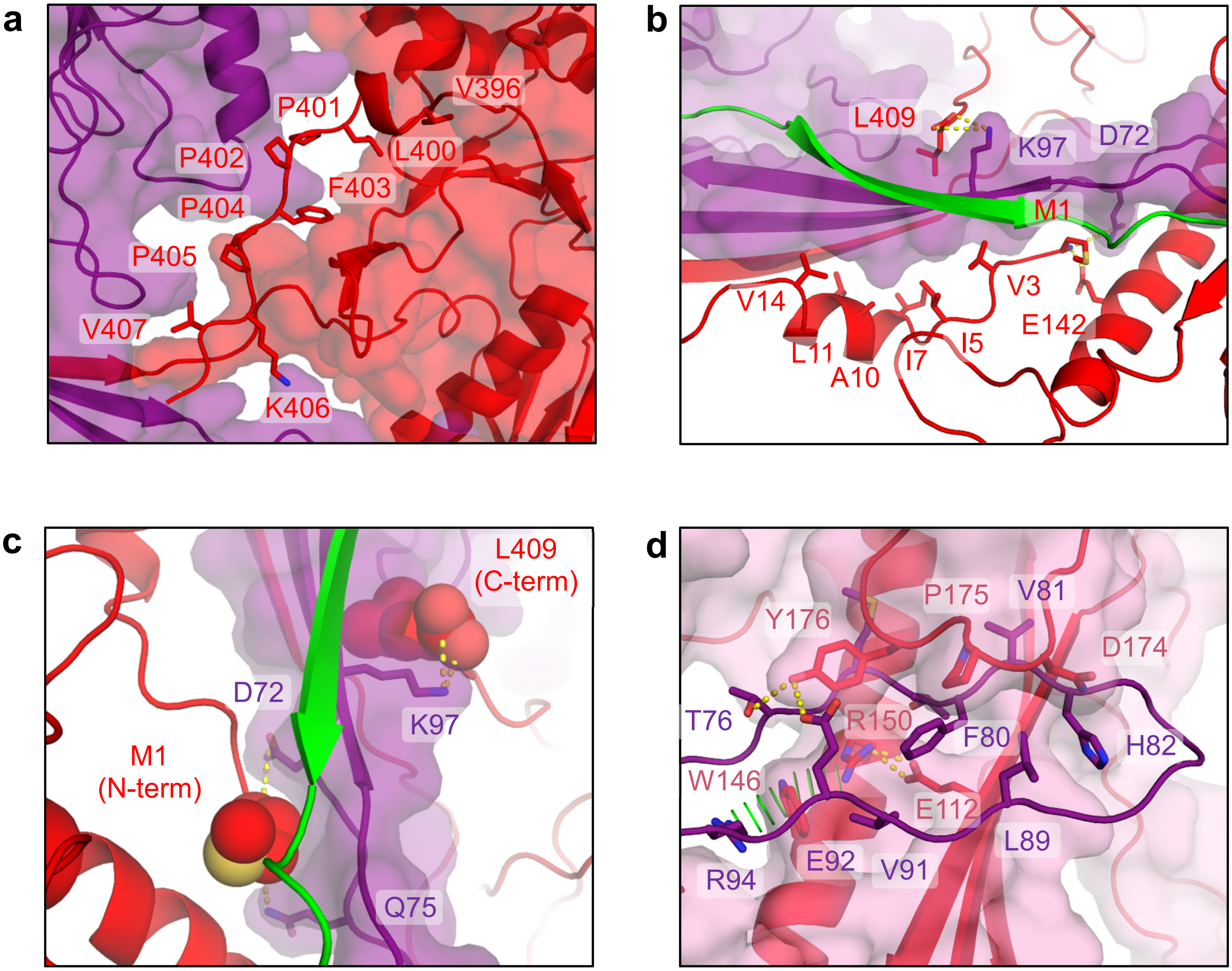

Supplementary Figure 6. Intra-capsomer interactions in P74-26.
a) The C-terminal arm forms hydrophobic interactions with both the A-domain of the MCP subunit from which it emanates (red), as well as an adjacent subunit (purple) stabilizing inter-capsomer interactions.
b) The MCP N- and C-termini (red) both interact with the E-loop β-sheet consisting of the E-loop (purple), N-arm (red) and Dec-arm (green). The C-arm interacts on the top of the β-sheet, while the N-arm binds along the bottom. Thus, the two arms collaborate to form a pincer-like interaction to hold the neighboring E-loop in place.
c) The MCP N- and C-termini (shown as spheres) use ionic interactions to act as pincers clasping the E-loop β-sheet from either side.
d) Hydrophobic interactions, hydrogen bonds, and salt bridges tightly secure the E-loop (purple) to the P-domain and G-loop of a neighboring subunit (red).

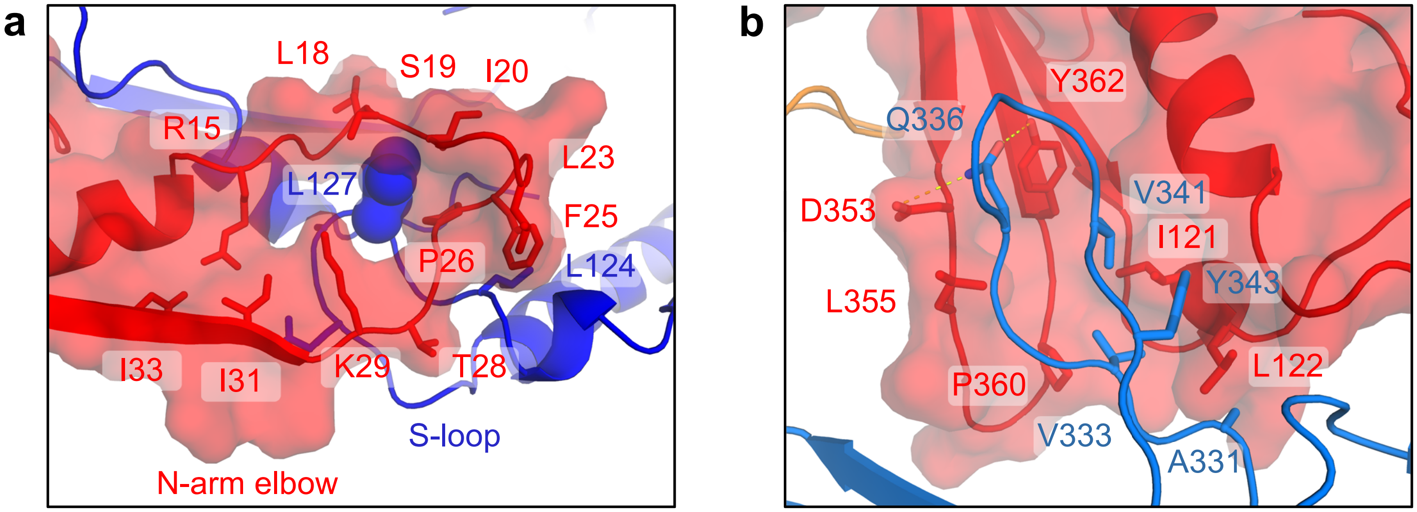

Supplementary Figure 7. Flap interactions across the two- and three-fold axes.
a) Leu 127 from the P-domain latch (dark blue) acts as a hitching post for the N-arm lasso of a neighboring MCP subunit (red) across the quasi-two-fold/two-fold axes.
b) The tongue-in-groove interaction of the MCP T-loop (light blue) and an adjacent MCP P-domain (red) at the quasi-three-fold/three-fold axes.

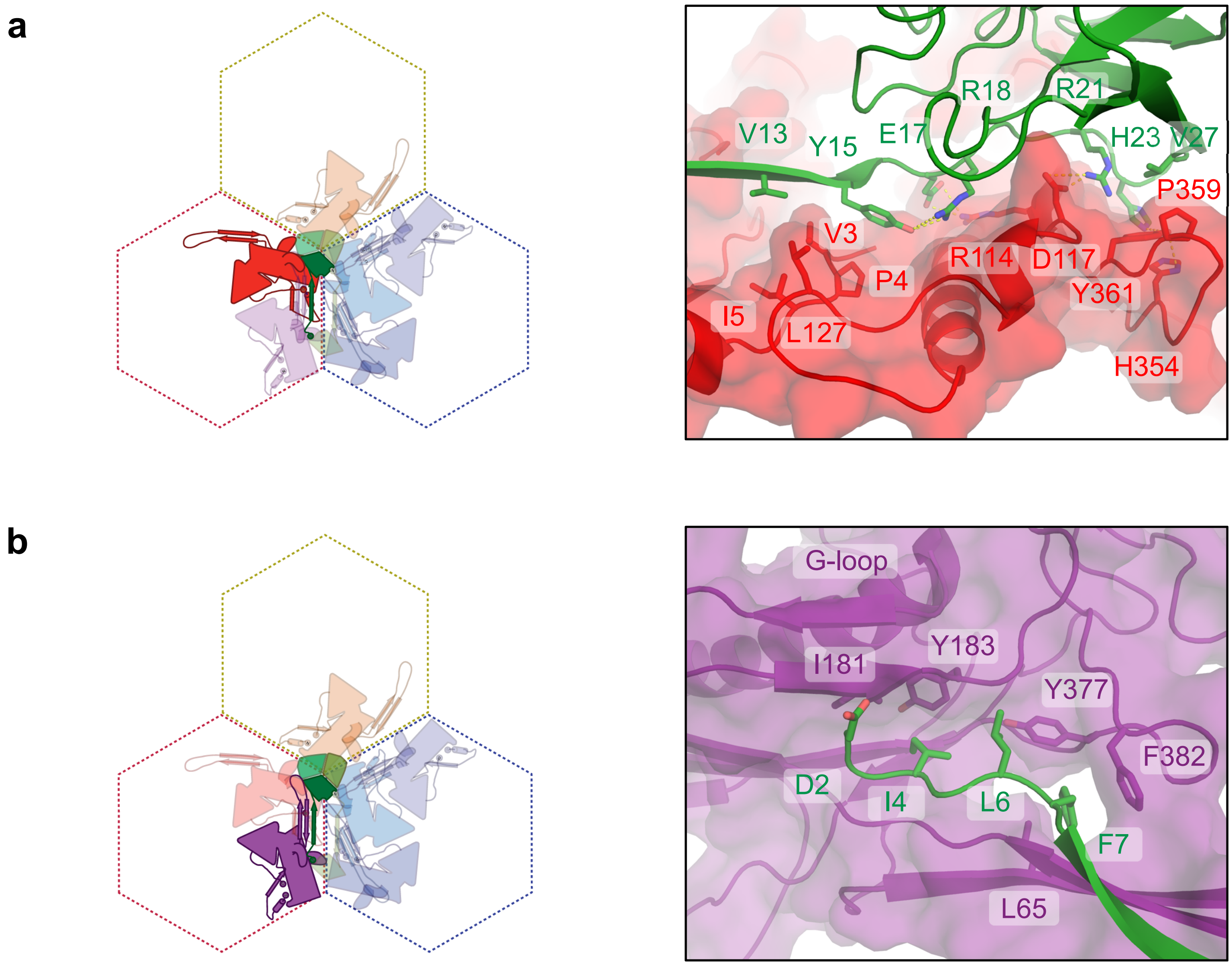

Supplementary Figure 8. Inter-capsomer interactions of Dec with α capsomer in P74-26.
a) The β-tulip domain of Dec (green) interacts extensively with the P-domain of the α1 subunit (red).
b) The Dec-arm (green) extends beyond the E-loop β-sheet, interacting with the G-loop and A-domain of the α2 subunit (purple).

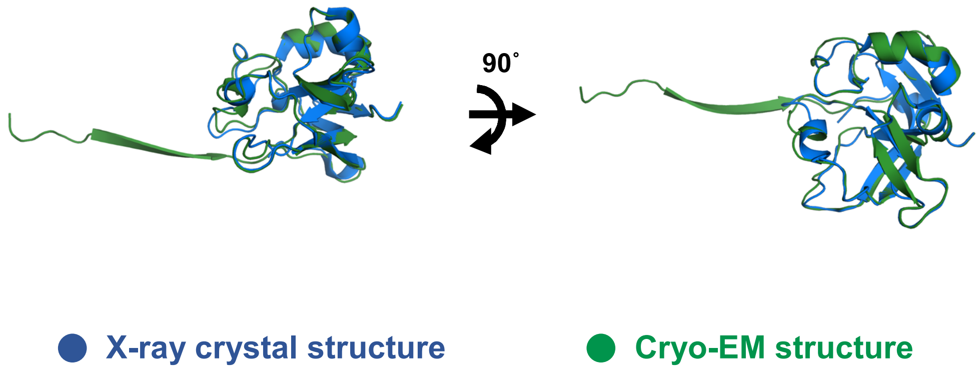

Supplementary Figure 9. Cryo-EM reconstruction of Dec^{P74-26}.
Comparison of the Dec^{P74-26} protein x-ray crystal structure (blue, PDB:6BL5) and the Dec^{P74-26} structure determined here by cryo-EM (green).

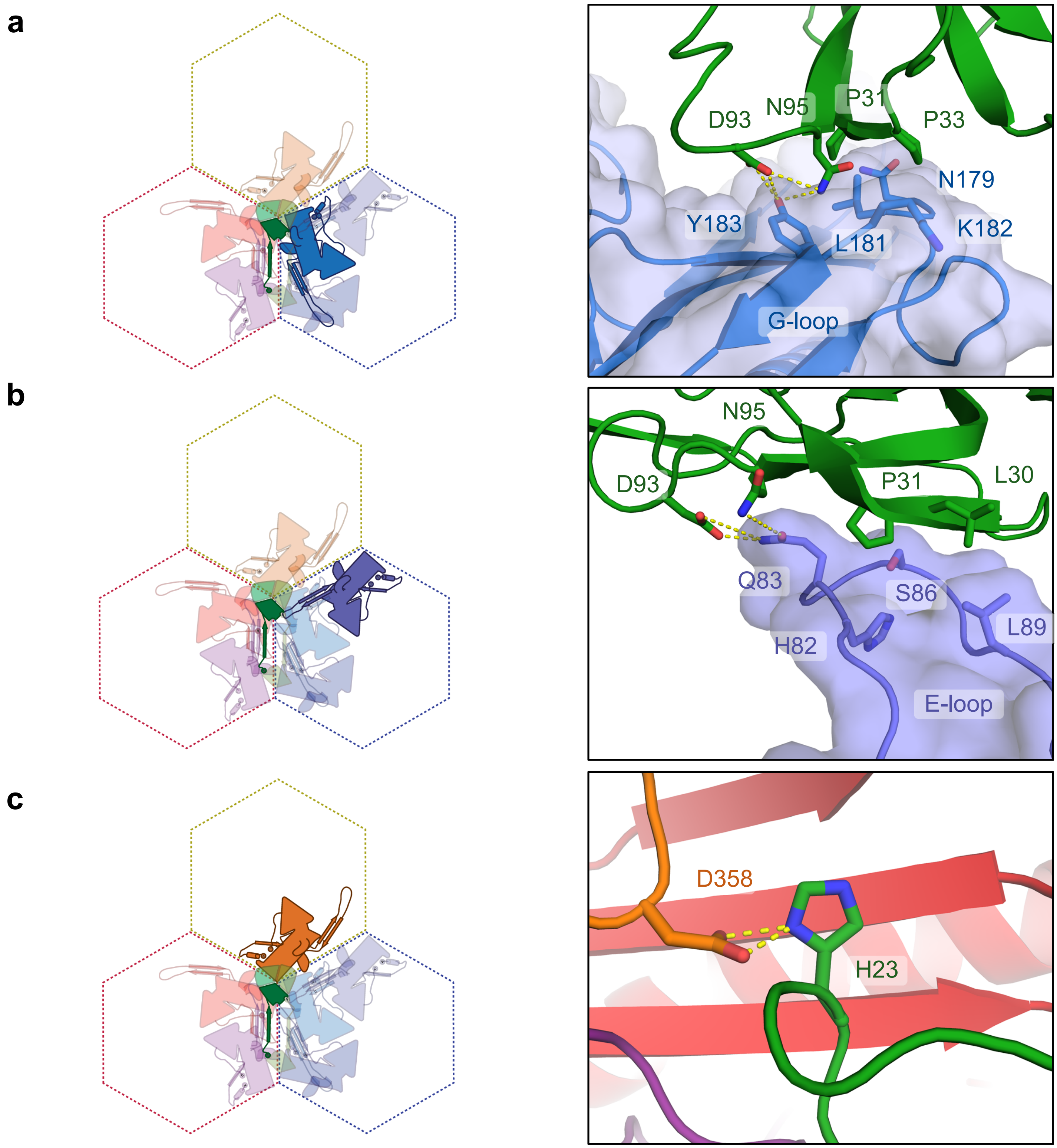

Supplementary Figure 10. Inter-capsomer interactions of Dec with β and γ capsomers in P74-26.
a) The Dec β-tulip domain (green) interacts with the extended G-loop of the β2 MCP subunit (blue) across the quasi-two-fold/two-fold axis.
b) The β-tulip domain of Dec (green) interacts with the tip of the E-loop of the β3 subunit (lavender).
c) The β-tulip domain of Dec (green) extends across the quasi-three-fold/three-fold axis, hydrogen bonding with the P-domain of the γ1 subunit (orange).

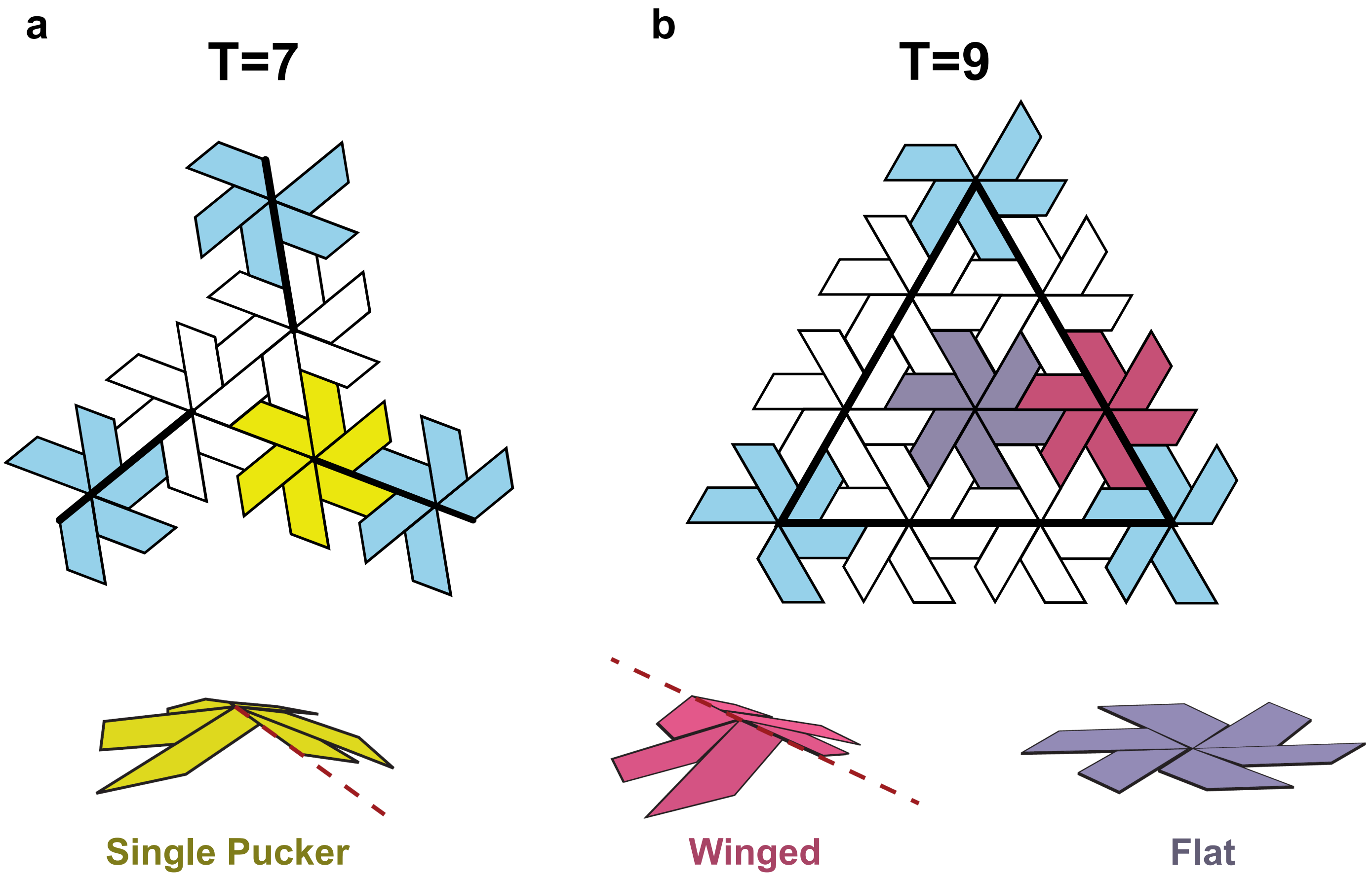

Supplementary Figure 11. Inter-capsomer interactions of Dec with β and γ capsomers in P74-26.
a and b) Schematic representation of the faces of a T=7 (A) and T=9 (B) icosahedron. T=7 viruses consist of equivalent pentons as well as equivalent hexons, which adopt a single pucker conformation. In T=9 viruses, two hexon conformations, winged and flat are required to form the larger capsid assembly. *Adapted from Mannige, et al, PLoS One, 2010.*

Supplementary Table 1: Structure determination and refinement

**Deposited structures**	
PDB accession no.	6O3H
EMDB accession no.	EMD-0618
**Data collection**	
Microscope	FEI Titan Krios
Detector	Gatan K-2
Voltage (kV)	300
Magnification	130,000
Electron exposure ($e^-/Å^2$)	48
Defocus range (μm)	-0.2 to -1.2
Pixel size (Å)	0.529
**Data processing**	
Number of particles refined	28,880
Final number of particles	23,178
Imposed symmetry	Icosahedral
Map-sharpening B-factor ($Å^2$)	-100
Final resolution (Å)	2.8
**Asymmetric unit refinement**	
Map correlation (%)	80.1
R.M.S.D. (bonds)	0.01
R.M.S.D. (angles)	0.93
All-atom clashscore	5.79
Ramachandran favored (%)	92.2
Ramachandran allowed (%)	7.3
Ramachandran Outliers (%)	0.49
Rotamer outliers (%)	0.42
C-beta deviations	0

Supplementary Table 2: Sizes of T=7 phage

Virus	Outer Diameter (Å) †	Inner Diameter (Å) ‡	Genome Size (kb)	Decoration Proteins	Packaging density (bp nm^{-3})	PDB/EMDB accession code
80α	618	488	43.9	none	0.72	6C21
ATCC_Clear	620	518	29.7•	none	0.41	3JB5
BPP-1	676	526	42.5	2-fold	0.56	3J4U
CW02	670	550	49.4	3-fold	0.57	3J1A
HK97	652	520	39.7	none	0.54	1OHG
Lambda	683	547	48.5	3-fold	0.57	EM-5012
P22	686	534	41.7	none	0.52	5UU5
P-SSP7	654	508	45	none	0.66	2XD8
Sf6	690	532	39	none	0.49	5L35
SPP1	676	556	45.9	hexon center	0.51	4AN5
SYN5	664	538	46.2	none	0.57	4BML
T7	664	522	39.9	none	0.54	3J7X
TW-1	700	558	39.9	3-fold	0.44	5WK1
ε15	668	536	39.7	2-fold	0.49	3C5B
HSTV-2§	738	608	68.2	3-fold	0.58	EM-2235
**P74-26**	**824**	**673**	**83.3**	**3-fold**	**0.52**	**6O3H**

† Outer diameter is defined as twice the length of the longest radius from the map center to the capsid shell.

‡ Inner diameter is defined as twice the length of the shortest radius from the map center to the capsid shell.

• Genome length reported from homolog propionibacterium phage PA6.
§ HSTV-2 is an archaeal virus.

Supplementary Table 3: Analysis of inter-subunit interactions across T=7 phages

Phage	MCP length (residues)	Average interface area (Å^2)†	$\Delta G_{hydrophobic}$ (kcal mol^{-1}) ‡	N_{HB}•	N_{SB}§	Structure resolution (Å)	PDB accession code
P74-26	409	3150	-34.1	34.3	3.8	2.8	6O3H
HK97	282	2346	-22.3	31.8	9.5	3.4	1OHG
Sf6	423	3277	-31.9	36.2	4.8	2.9	5L35
TW1	352	2449	-21.4	30.2	5.3	3.6	5WK1
P22	430	3230	-24.4	41.8	9.3	3.3	5UU5
BPP-1	331	2416	-18.4	27.0	12.5	3.5	3J4U
T7	398	2861	-29.9	25.3	3.8	3.5	3J7W
ATTC_Clear	315	2226	-26.6	22.8	1.5	3.7	3JB5

† Calculated as difference in total accessible surface areas of isolated and interfacing subunits within a hexon divided by two, averaged over all six hexon interfaces.

‡ Average estimated hydrophobic interaction energy between two subunits within a hexon, averaged over all six hexon interfaces. $\Delta G_{hydrophobic}$ indicates the solvation free energy gain upon formation of the interface, in kcal mol^{-1}. The value is calculated as difference in total solvation energies of isolated and interfacing structures.

• Number of hydrogen bonds between two subunits within a hexon, averaged over all six hexon interfaces.

§ Number of salt bridges between two subunits within a hexon, averaged over all six hexon interfaces.

Supplementary Table 4: Analysis of decoration protein interactions in P74-26 and phage TW1

Phage	Dec length (residues)	Average interface area ($Å^2$)†	$\Delta G_{hydrophobic}$ ($kcal\ mol^{-1}$) ‡	N_{HB} •	N_{SB} §	No. of proteins contacted	Structure resolution (Å)	PDB accession code
P74-26	146	4162	-38	41	24	9	2.8	6O3H
TW1	149	2676	-9	34	13	7	3.6	5WK1

† Calculated as the total interaction surface of a decoration protein monomer with adjacent MCP and Dec subunits.

‡ Total estimated hydrophobic interaction energy between Dec and binding partners, averaged from three Dec proteins within Dec trimer. $\Delta G_{hydrophobic}$ indicates the solvation free energy gain upon formation of the interface, in $kcal\ mol^{-1}$. The value is calculated as difference in total solvation energies of isolated and interfacing structures.

• Total number of hydrogen bonds between Dec and interacting partners, averaged from three Dec proteins within trimer.

§ Number of salt bridges between Dec monomer and interacting proteins, averaged from three Dec proteins within trimer.
