# Supplementary material for: Principles for enhancing virus capsid capacity and stability from a thermophilic virus capsid structure
Source: Nat Commun. 2019 Oct 2;10:4471. doi: 10.1038/s41467-019-12341-z (PMC6775164; doi:10.1038/s41467-019-12341-z)
Supplement: Supplementary file 3 — Reporting Summary [file 41467_2019_12341_MOESM3_ESM.pdf]

nature research

Corresponding author(s): Brian A Kelch

Last updated by author(s): Aug 16, 2019

Reporting Summary

Nature Research wishes to improve the reproducibility of the work that we publish. This form provides structure for consistency and transparency in reporting. For further information on Nature Research policies, see Authors & Referees and the Editorial Policy Checklist .

Statistics

For all statistical analyses, confirm that the following items are present in the figure legend, table legend, main text, or Methods section.

n/a	Confirmed	
☐	☒	The exact sample size (n) for each experimental group/condition, given as a discrete number and unit of measurement
☐	☒	A statement on whether measurements were taken from distinct samples or whether the same sample was measured repeatedly
☒	☐	The statistical test(s) used AND whether they are one- or two-sided<br>*Only common tests should be described solely by name; describe more complex techniques in the Methods section.*
☒	☐	A description of all covariates tested
☒	☐	A description of any assumptions or corrections, such as tests of normality and adjustment for multiple comparisons
☒	☐	A full description of the statistical parameters including central tendency (e.g. means) or other basic estimates (e.g. regression coefficient) AND variation (e.g. standard deviation) or associated estimates of uncertainty (e.g. confidence intervals)
☒	☐	For null hypothesis testing, the test statistic (e.g. F, t, r) with confidence intervals, effect sizes, degrees of freedom and P value noted<br>*Give P values as exact values whenever suitable.*
☒	☐	For Bayesian analysis, information on the choice of priors and Markov chain Monte Carlo settings
☒	☐	For hierarchical and complex designs, identification of the appropriate level for tests and full reporting of outcomes
☒	☐	Estimates of effect sizes (e.g. Cohen's d, Pearson's r), indicating how they were calculated

Our web collection on statistics for biologists contains articles on many of the points above.

Software and code

Policy information about availability of computer code

Data collection	The SerialEM software suite and the Gatan Digital Micrograph suite were used to collect cryoelectron microscopy and negative-stain microscopy images, respectively.
Data analysis	cisTEM version 1.0 and Frealign version 9.11 were used for processing, classification, and 3D refinement of electron microscopy data. Coot version 0.8 was used for atomic model building of the asymmetric unit model. The Phenix software suite was used for real-space refinement of the final atomic model.

For manuscripts utilizing custom algorithms or software that are central to the research but not yet described in published literature, software must be made available to editors/reviewers. We strongly encourage code deposition in a community repository (e.g. GitHub). See the Nature Research guidelines for submitting code & software for further information.

Data

Policy information about availability of data

All manuscripts must include a data availability statement. This statement should provide the following information, where applicable:

- Accession codes, unique identifiers, or web links for publicly available datasets
- A list of figures that have associated raw data
- A description of any restrictions on data availability

Model coordinates of the P74-26 capsid asymmetric unit were deposited to the Protein Data Bank (PDB: 6O3H). The density map of the icosahedral capsid reconstruction was deposited to the EM Data Bank (EMD-0618).

Field-specific reporting

Please select the one below that is the best fit for your research. If you are not sure, read the appropriate sections before making your selection.

- [x] Life sciences
- [ ] Behavioural & social sciences
- [ ] Ecological, evolutionary & environmental sciences

For a reference copy of the document with all sections, see nature.com/documents/nr-reporting-summary-flat.pdf

Life sciences study design

All studies must disclose on these points even when the disclosure is negative.

Sample size	The data presented in Figure 1B is collated from three independent experiments, and error bars represent the standard deviation between these experiments. Three independent experiments were sufficient to calculate an inhibition curve for the phage infectivity assay.
Data exclusions	No data were excluded from the statistical data shown in the manuscript.
Replication	All attempts at validating and repeating the infectivity assay in Figure 1B were successful.
Randomization	Randomization was not relevant to the given study, as the experimental data was not subdivided into different experimental groups.
Blinding	Blinding was not relevant to the given study as no information from the experimental design described would bias the results.

Reporting for specific materials, systems and methods

We require information from authors about some types of materials, experimental systems and methods used in many studies. Here, indicate whether each material, system or method listed is relevant to your study. If you are not sure if a list item applies to your research, read the appropriate section before selecting a response.

Materials & experimental systems

n/a	Involved in the study	
x		Antibodies
x		Eukaryotic cell lines
x		Palaeontology
x		Animals and other organisms
x		Human research participants
x		Clinical data

Methods

n/a	Involved in the study	
x		ChIP-seq
x		Flow cytometry
x		MRI-based neuroimaging
